# Frequency of Specific Cardiovascular Disease Risk Factors among Cameroonian Patients on Dialysis: The Cases of Anaemia, Inflammation, Phosphate, and Calcium

**DOI:** 10.1155/2016/5031927

**Published:** 2016-05-22

**Authors:** Olivier Pancha Mbouemboue, Olivier Djile Danbe, Marcel Tangyi Tamanji, Jacques Olivier Ngoufack

**Affiliations:** ^1^Department of Biomedical Sciences, Faculty of Science, University of Ngaoundéré, P.O. Box 454, Ngaoundéré, Cameroon; ^2^General Medicine Service, Ngaoundéré Regional Hospital, P.O. Box 45, Ngaoundéré, Cameroon; ^3^Clinical Laboratory Service, Ngaoundéré Regional Hospital, P.O. Box 45, Ngaoundéré, Cameroon; ^4^Faculty of Science, University of Buea, P.O. Box 63, Buea, Cameroon

## Abstract

Specific cardiovascular risk factors are known to contribute to increasing cardiovascular mortality in patients with chronic renal disease. However, little is known about their distribution in our population. This study aimed at determining the prevalence of anaemia, inflammation, and phosphocalcium disorders in Cameroonian patients on dialysis. Thirty-five participants with stage V chronic kidney disease (defined by glomerular filtration rate, GFR < 15 mL/1.73 m^3^) of age at least 20 years on haemodialysis were randomly recruited. A control group composed of persons without a history of renal or cardiovascular disease was also recruited. Haemoglobin concentration, serum phosphate concentration, serum calcium concentration, and CRP status as a marker of inflammation were determined for all participants. Anaemia, phosphocalcium metabolic disorders, and a positive CRP result among haemodialysed patients were estimated at 94.3%, 61.6%, and 77.1%, respectively. Anaemia was diagnosed in all female patients compared to 92% in males, while a positive CRP result was recorded in 90% of females and 72% of males. No significant differences were observed on the distribution of studied specific cardiovascular risk factors with duration of dialysis. Among the factors studied, anaemia was the most encountered.

## 1. Introduction

Several studies have indicated that the traditional cardiovascular disease risk factors (CVDRF) alone do not explain the excess cardiovascular disease (CVD) risk in patients with ESRD. It has been shown that nontraditional risk factors for CVDs increase the mortality rate in patients with End Stage Renal Disease (ESRD) 10- to 20-fold compared to the general population [1]. These factors include anaemia, phosphocalcium disorders, inflammation, oxidative stress, hypoalbuminaemia, and hyperhomocysteinaemia. Although these factors are identified, little is known about their distribution in our population. In this work, we studied some of these factors with the goal of determining their distribution among patients on haemodialysis in our context.

## 2. Materials and Methods

This cross-sectional study was carried at the Haemodialysis Center of the Garoua Regional Hospital from April to August 2014 in which participants were randomly recruited. A control group composed of persons without any history of renal or cardiovascular disease were likewise recruited. The biological variables of interest in this study included haemoglobin, C-reactive protein (CRP), and calcium and phosphate levels in blood.

### 2.1. Inclusion and Exclusion Criteria

Patients with stage V chronic kidney disease (CKD) (defined by glomerular filtration rate, GFR < 15 mL/1.73 m^3^) of age at least 20 years and on haemodialysis were enrolled while those younger than 20 years old were excluded.

### 2.2. Laboratory Measurements

#### 2.2.1. Determination of Haemoglobin Concentration

Haemoglobin concentrations were determined using Mindray BC-3200 auto haematology analyser (Marburg, Germany), which measures haemoglobin by colorimetry as described by Lewis (2002) [2].

#### 2.2.2. Determination of Biochemical Markers

Biochemical analyses were carried out using a semi-auto chemistry analyser (SECOMAN Basic/70V B0358, SN: 1790, France). Prior to analyses, the spectrophotometer was calibrated with the help of a Calimat calibrator (reference: 62321, Lot: 1003001280 Biomérieux, France).

*(1) Serum Calcium Concentration*. Calcium measurements were carried out by spectrophotometry using commercial kit based on the Arsenazo III method described by Endres and Rude in 1999 [3]. The reference values were indicated as 82–108 mg/L.

*(2) Serum Phosphate Concentration*. Measurement of serum phosphate levels was by spectrophotometry using commercially acquired kits based on the ammonium molybdate method previously described by Endres and Rude [3]. Reference values were considered within the range 25–45 mg/L.

#### 2.2.3. C-Reactive Protein Detection

CRP status, as a marker of inflammation, was qualitatively determined using a rapid CRP latex agglutination test, and results were reported as positive or negative.

### 2.3. Quality Assurance

The use of appropriate glass- and plasticware and calibrated micropipettes was implemented. R verification and validation of results was done using a Lyotrol N quality control serum (ref: 62373, Lot: 1003001280, Biomérieux, France) and ABX Minotrol 16 haematology control (ref: 2042002, Lot: MX114TN, France) for serum phosphocalcium and haemoglobin analyses, respectively. Results were considered valid when control values were within mean ± 2SD and in conformity with Westgard's rules.

### 2.4. Data Analysis

Data were analysed using the statistical package for social sciences (SPSS) version 20 software and results on prevalence were expressed as percentages. The Chi-square and ANOVA test were used to compare occurrence rates and means between populations, and statistical significance was considered at *p* < 0.05.

### 2.5. Ethical Considerations

This study was granted the approval of the Garoua Regional Hospital. Patients were adequately informed about the aim, merits, and demerits of this study and consented to participate therein. Participants' information was confidentially protected and they were free to discontinue from the study at any time they pleased without any sanctions.

## 3. Results

Thirty-five CKD patients were in conformance with the stipulated inclusion criteria, comprising 25 men and 10 females and having an average age of 45 ± 14.6 years. Following the outcomes of this study, patients undergoing haemodialysis recorded significantly lower mean haemoglobin and calcium levels and increased phosphate concentrations compared to healthy controls. Also, the prevalence of a positive CRP result was higher among study participants when compared to the controls (Table 1).

Anaemia, phosphocalcium metabolic disorders, and a positive CRP result among haemodialysed patients were estimated at 94.3%, 61.6%, and 77.1% respectively.

Gender based distribution of specific CVDRF among CKD on dialysis is represented in Table 2.

Anaemia was diagnosed in all female patients compared to 92% in males, while a positive CRP result was recorded in 90% of females and 72% of males. No significant differences were observed between male and female participants following comparison of the mean levels of haemoglobin (9.27 ± 1.53 versus 8.98 ± 0.63 mg/dL; *p* = 0.054), phosphate (69.2 ± 26.62 versus 68.6 ± 25.3 mg/L; *p* = 0.894), and calcium (74.48 ± 15.41 versus 62.2 ± 21.12 mg/L; *p* = 0.065). Moreover, all patients aged 50 years and above presented with anaemia, while anaemia was observed in 88.8% of participants under 50 years of age (*p* = 0.22).

Furthermore, a positive CRP result was recorded in 82.3% of participants aged 50 years and above and 72.2% of those under 50 years of age (*p* = 0.202). The mean haemoglobin, phosphate, and calcium levels in patients under 50 years of age compared to those of 50 years and above were 9.3 ± 1.55 g/dL versus 8.64 ± 1.18 g/dL, 69.39 ± 26.40 mg/L versus 69.71 ± 26.13 mg/L, and 74.67 ± 15.06 mg/L versus 67.06 ± 20.05 mg/L, respectively. No statistically significant differences were observed on the distribution of specific CVDRF between age groups (Table 3).

Anaemia was observed in about half of the participants who had been on dialysis for 1–5 years (54.5%) but in only 30.3% of participants who had been for less than a year, although participants in the early stage of dialysis (<1 year) recorded lower mean haemoglobin levels compared to those with a longer duration (≥1 year) (Table 4). Conversely, the mean phosphate and calcium levels were almost identical between the groups of participants with varying duration of dialysis. No significant differences were observed on the distribution of CVDRF with duration of dialysis.

## 4. Discussion

The occurrence of cardiovascular diseases worldwide continues to increase and in addition to well documented conventional risk factors, other modifiable nontraditional risk factors further complicate clinical management of affected persons.

### 4.1. Anaemia

Anaemia is a common complication and predicts mortality in patients on haemodialysis [4]. The physiopathology of anaemia associated with CKD can be explained by two mechanisms: firstly, a decrease in erythropoiesis owing to a decrease in the production and secretion of erythropoietin (EPO) and secondly the occurrence of a disorder of iron metabolism [5]. Anaemia is considered as a novel cardiovascular disease risk which mediates a cardiac function overload resulting in a left ventricular hypertrophy (LVH), with the occurrence and progression of LVH corroborating with anaemia severity and associated with increased mortality risk [6].

In our study, the quasi total of haemodialysed (94.3%) patients was anaemic. In a study by Afshar and colleagues, the prevalence of anaemia among haemodialysed patients was 85% [6]. A lower prevalence was reported by Brunelli and Berns [7]. The increased prevalence observed in our study could be explained by the absence of preventive anaemia management in our patients. It has been well established that the occurrence of anaemia increases with the stage of CKD and tends to correlate with creatinine clearance [8]. In line with the study of Rotan Das Gupta et al., patients undergoing haemodialysis recorded a lower mean haemoglobin level compared to healthy controls.

### 4.2. Inflammation

C-reactive protein is an acute phase reactant and a positive result represents a marker of inflammation. 30 to 50% of patients on dialysis present increased levels of an inflammatory marker. The presence of chronic or episodic inflammation has been proven to be associated with increased risk of death [9]. Following literature, the causes of inflammation are multifactorial and on the one hand include renal disease, comorbidities, oxidative stress, infections, obesity, genetic and immunologic factors, and other factors associated with haemodialysis [10]. Several studies have reported an increased prevalence of CRP in haemodialysed populations [11, 12]. In the present study, a positive CRP was found in 67% of haemodialysis patients against 14.3% in the control group.

### 4.3. Phosphocalcium Metabolic Disorders

Hypocalcaemia is frequently associated with hyperphosphataemia in CKD with its mechanism associated with primary hyposecretion of parathormone which results in a progressive decrease in blood calcium levels up to a steady state characterised by an equality between urinary calcium excretion and osseous calcium secretion [13, 14].

In this study, hyperphosphataemia and hypocalcaemia were recorded at 88.6% and 71.1%, respectively, with 61.6% of study participants presenting with both. Abderraman et al. reported 30.8% hyperphosphataemia and 23% hypocalcaemia, suggesting that the overall prevalence of phosphocalcic disorders was 53.8% in their study [15]. The mean phosphate level in our study was noted to be higher in haemodialysed participants (69.54 ± 25.88) when compared to controls (37.63 ± 4.72). This is supported by the findings of Karimi et al. who recorded a net hyperphosphoraemia in ESRD patients on dialysis [16].

*Limitations and Perspectives*. Several other novel risk factors have not been explored due to technical drawbacks and the high cost of laboratory-based tests. The use of the qualitative latex agglutination in the determination of CRP compared to the high sensitive CRP (hsCRP) was a major setback in this study as the frequency of a positive CRP result was subjected to possible underestimation and the degree of inflammation was not assessed. Moreover, this was a pioneer study in our resource limited environment characterised by a low level of awareness on renal failure and the need for haemodialysis procedures, resulting in a small sample size which limits the extrapolation of our findings to a larger population. Further studies should be oriented towards novel risk factors in ESRD patients on dialysis in our environment in a bid to provide more clinical and epidemiologic information necessary for their management.

## 5. Conclusion

This study confirms the extraordinarily high rates of anaemia, inflammation, and phosphocalcium metabolic disorders in stage V chronic kidney disease patients on dialysis. Regarding these emerging risk factors, anaemia recorded the highest frequency in our study population.

Our study also reveals that these specific cardiovascular risk factors equivalently occur in hemodialysed patients without differences with respect to gender, age, and duration of dialysis.

## Figures and Tables

**Table 1:** Comparison of specific cardiovascular disease risk factors between haemodialysed patients and healthy controls.

Variables	Patients		Control		*p* value
	*N*	%	*N*	%	
(1) Anaemia					
No	2	5.7	28	82.4	0.001
Yes	33	94.3	6	17.6	

(2) CRP					
Positive	27	77.1	5	14.3	0.001
Negative	8	22.9	30	85.7	

(3) Mean values (Mean ± SD)					
Haemoglobin (g/dL)	8.98 ± 1.40		13.93 ± 1.50		0.001
Phosphate (mg/L)	69.54 ± 25.88		37.63 ± 4.72		0.001
Calcium (mg/L)	70.97 ± 17.82		92.14 ± 6.42		0.001

**Table 2:** Distribution of specific cardiovascular disease risk factors among haemodialysed patients by gender.

Variables	Males		Females		Total		*p* value
	*N*	%	*N*	%	*N*	%	
(1) Anaemia							
No	2	8	—	—	2	5.7	0.504
Yes	23	92	10	100	33	94.2	

(2) CRP							
Positive	18	72	9	90	27	77.1	0.250
Negative	7	28	1	10	8	22.8	

(3) Mean values (Mean ± SD)							
Haemoglobin (g/dL)	9.27 ± 1.53		8.26 ± 0.63		8.98 ± 1.40		0.054
Phosphate (mg/L)	69.2 ± 26.62		68.6 ± 25.3		69.54 ± 25.88		0.894
Calcium (mg/L)	74.48 ± 15.41		62.2 ± 21.12		70.57 ± 17.82		0.065

**Table 3:** Distribution of specific cardiovascular disease risk factors among haemodialysed patients by age.

Variables	20–49		≥50		Total		*p* value
	*N*	**%**	*N*	%	*N*	**%**	
(1) Anaemia							
No	2	11.2	—	—	2	5.8	0.220
Yes	16	88.8	17	100	33	94.2	

(2) CRP							
Positive	13	72.2	14	82.3	27	77.1	0.202
Negative	5	27.7	3	17.6	8	23.3	

(3) Mean values (Mean ± SD)							
Haemoglobin (g/dL)	9.3 ± 1.55		8.64 ± 1.18		8.98 ± 1.40		0.054
Phosphate (mg/L)	69.39 ± 26.40		69.71 ± 26.13		69.54 ± 25.88		0.894
Calcium (mg/L)	74.67 ± 15.06		67.06 ± 20.05		70.97 ± 17.82		0.065

**Table 4:** Distribution of specific cardiovascular disease risk factors among study participants by duration of dialysis.

Variables	Duration of dialysis						
	<1 year		1–5 years		5–10 years		*p* value
	*N*	**%**	*N*	**%**	*N*	**%**	
(1) Anaemia							
No	2	5.7	—	—	—	—	0.131
Yes	10	30.3	18	54.5	5	15.2	

(2) CRP							
Positive	9	33.3	13	48.1	5	18.5	0.415
Negative	3	37.5	5	31.3	—	—	

(3) Mean values (Mean ± SD)							
Haemoglobin (g/dL)	8.36 ± 1.57		9.32 ± 1.29		9.24 ± 1.05		0.173
Phosphate (mg/L)	71.67 ± 24.8		71.89 ± 29.67		56.00 ± 12.00		0.210
Calcium (mg/L)	70.33 ± 16.00		67.83 ± 19.81		83.80 ± 8.67		0.463

## References

[1] Zumrutdal A. (2011). Determinants of cardiovascular risk in hemodialysis patients without significant comorbidities. *Progress in Hemodialysis—From Emergent Biotechnology to Clinical Practice*.

[2] Lewis M. (2002). Anaemia and haemoglobinometry in rural areas. *Africa Health*.

[3] Endres D. B., Rude R. K., Burtis C. A., Ashwood E. R. (1999). Mineral and bone metabolism. *Tietz Textbook of Clinical Chemistry*.

[4] Lankhorst C. E., Wish J. B. (2010). Anemia in renal disease: diagnosis and management. *Blood Reviews*.

[5] Zadrazil J., Horak P. (2015). Pathophysiology of anemia in chronic kidney diseases: a review. *Biomedical Papers*.

[6] Afshar R., Sanavi S., Salimi J., Ahmadzadeh M. (2010). Hematological profile of Chronic Kidney Disease (CKD) patients in Iran, in pre-dialysis stages and after initiation of hemodialysis. *Saudi Journal of Kidney Diseases and Transplantation*.

[7] Brunelli S. M., Berns J. S. (2009). Anemia in chronic kidney disease and end-stage renal disease. *Nephrology Rounds*.

[8] Stauffer M. E., Fan T. (2014). Prevalence of anemia in chronic kidney disease in the United States. *PLoS ONE*.

[9] Nusair M. B., Rajpurohit N., Alpert M. A. (2012). Chronic inflammation and coronary atherosclerosis in patients with end-stage renal disease. *Cardiorenal Medicine*.

[10] Bazeley J., Bieber B., Li Y. (2011). C-reactive protein and prediction of 1-year mortality in prevalent hemodialysis patients. *Clinical Journal of the American Society of Nephrology*.

[11] Razeghi E., Omati H., Maziar S., Khashayar P., Mahdavi-Mazdeh M. (2008). Chronic inflammation increases risk in hemodialysis patients. *Saudi Journal of Kidney Diseases and Transplantation*.

[12] Nakayama M., Ura Y., Nagata M. (2011). Carotid artery calcification at the initiation of hemodialysis is a risk factor for cardiovascular events in patients with end-stage renal disease: a cohort study. *BMC Nephrology*.

[13] Block G. A., Ix J. H., Ketteler M. (2013). Phosphate homeostasis in CKD: report of a scientific symposium sponsored by the National Kidney Foundation. *American Journal of Kidney Diseases*.

[14] Block G. A., Klassen P. S., Lazarus J. M., Ofsthun N., Lowrie E. G., Chertow G. M. (2004). Mineral metabolism, mortality, and morbidity in maintenance hemodialysis. *Journal of the American Society of Nephrology*.

[15] Abderraman G. M., Ka E. F., Cisse M. M. (2015). Evaluation of phospho-calcic profile of dakar chronic hemodialysis and comparison with KDIGO recommendations. *International Journal of Nephrology and Kidney Failure*.

[16] Karimi I., Benabdellah N., Bentata Y., Yacoubi H., Haddiya I. (2015). Evaluation du niveau d'activité physique dans un service Marocain d'hémodialyse chronique. *Pan African Medical Journal*.

